# Comfort with LGB people and attitudes toward same-sex parenting in Continental American Hispanic Nations

**DOI:** 10.1038/s41598-024-56901-w

**Published:** 2024-04-02

**Authors:** Fernando Salinas-Quiroz, Julian H. Balkcom, Carlos Hermosa-Bosano, Adriana Olaya-Torres, Pedro Alexandre Costa

**Affiliations:** 1 Eliot-Pearson Department of Child Study and Human Development, Tufts University, Medford, USA; 2 School of Psychology and Education, Universidad de Las Américas, Quito, Ecuador; 3 Faculty of Humanities, Arts and Social Sciences, University of Ibagué, Ibagué, Colombia; 4 Faculty of Psychology and Education Sciences, University of Porto, Porto, Portugal

**Keywords:** Sexual prejudice, Interpersonal contact, Same-sex parented families, Homonegativity, LGBT individuals, Continental American Hispanic Nations (CAHN), Human behaviour, Developmental biology

## Abstract

Negative attitudes toward Lesbian, Gay, and Bisexual (LGB) individuals leads to a perceived inability of LGB individuals to foster ‘appropriate’ family relationships, inciting negative attitudes specifically toward same-sex parenting. Intergroup and interpersonal relationships play a critical role in fostering attitudes toward others wherein type of contact, frequency, degree of closeness in the relationship, and the positivity/negativity of interactions are potential mediator of these relations, Moreover, the mechanism behind co-constructing positive relationships with sexual and gender minorities is comfort with contact with LGB individuals. The present study explored the effects of interpersonal contact and the mediator role of comfort with LGB people in explaining attitudes toward same-sex parenting in Spanish-speaking countries in North, Central, and South America. These countries are of particular interest given the dearth of research in the region on attitudes toward same-sex parenting as well as the varying degrees of acceptance of and protections for same-sex parented families. A non-probabilistic sample of 1955 heterosexual cisgender participants from 14 countries was asked to complete a series of sociodemographic questions, a questionnaire about their interpersonal contact/comfort experiences with LGB people, and the Attitudes Toward Gay and Lesbian Parenting Scale. Results showed that comfort was vital in fostering accepting attitudes toward Same-Sex Parenting across countries. Findings also suggested that comfort with LGB people has a particularly powerful influence in regions with less legal and cultural acceptance of LGB individuals. Policies are not enough to instill widespread change: we must encourage, facilitate, and supervise the formation of relationships with LGB people.

## Introduction

The conceptualization of family has extended beyond a ‘traditional’ different-sex (heterosexual) couple and their children to be inclusive of individuals from diverse sexual identities and orientations as the pathways to parenthood are becoming increasingly accessible both in terms of social acceptance and in terms of legal frameworks throughout the westernized world, families headed by same-sex parents, particularly planned families through assisted reproductive technologies, donor insemination or adoption have become steadily visible^1^. However, as they claim their space in society, opposition to their legitimacy concurrently materializes, alleging that same-sex parents do not deserve identical rights to different-sex parents^2^. By exploring the attitudes of those against same-sex parenting that shape their prejudicial beliefs, we can attempt to understand the root of such resistance.

An individual’s attitude toward a person or a specific group encompasses their evaluative judgment^3^. Attitudes and judgments inform whether society deems a group to be worthy of equal status and treatment^4^. Homonegativity can be conceptualized as heterosexuals’ negative attitudes toward Lesbian, Gay, and Bisexual (LGB) individuals because of their sexual orientation^5^. Negative attitudes engender stigma toward a minoritized group, such as LGB people, which socially devalues them as inferior^4^. Consequently, the societal internalization of sexual stigma manifests prejudice against sexual minorities^4^. Heterosexism, or the societally-held notion that heterosexuals are superior to all sexual minorities^6^, helps maintain negative attitudes toward LGB people and same-sex parenting by rationalizing heterosexuals’ stigmatization of them. Compulsory heterosexuality, in addition, acts as a reinforcing mechanism that perpetuates societal norms favoring heterosexuality. Originally coined by Rich in 1980^7^, this concept highlights the social expectations that coerce individuals into conforming to heterosexuality and its associated norms, fostering an environment where alternative sexual and gender identities become marginalized and stigmatized. These social expectations are deeply ingrained in various cultural and social institutions, including the family, educational systems, healthcare, media representation, and the workplace. Consequently, those who deviate from the presumed heterosexual norm often face discrimination, prejudice, and unequal opportunities^8^. Sexual prejudice, heterosexism and compulsory heterosexuality thus contribute to the suppression of LGB equality and the maintenance of sexual and gender minorities’ inferior status^9^.

Historically, homonegative attitudes toward LGB people and same-sex parenting may be rooted in their perceived inability to foster safe, healthy, and ‘appropriate’ family relationships and dynamics, inciting negative attitudes specifically toward same-sex parenting^9–11^. In a normative family that believes in and reproduces traditional gender norms and compulsory heterosexuality, women are expected to assume the ‘feminine role’ of caregiving within the family and home, whereas the man’s role of leadership, working outside of the home, aligns with masculine social norms^12,13^. However, LGB individuals are thought as violating these gender roles in their parenting, since fervent opposers to same-sex parented families allege that they model inappropriate gender roles due to the absence of ‘the opposite gender’^12,13^. Same-sex parents are also believed to expose their children to bullying and harassment, and influence them to becoming gender-nonconforming or a sexual and/or gender minority themselves^14,15^. On the contrary, literature on the parenting abilities of LGB caregivers^16,17^ and on their children’s psychological and social adjustment^18,19^ consensually shows that LGB parented families fare at least as well as their heterosexual counterparts. Thus, the stereotypes that shape negative attitudes toward same-sex parenting appear to be untrue and detrimental.

Attitudes toward individuals and groups play a critical role in shaping intergroup and interpersonal relationships. In 1954, Gordon Allport devised the contact hypothesis, which posits that reducing an in-group’s prejudice toward a given out-group can be achieved by building interconnections between them. The concept of *familiarity becomes liking* governs this theory: as evident in longitudinal studies, more frequent contact can decrease intergroup prejudice^20,21^. In a meta-analysis, Smith et al.^22^ found that increased contact with lesbian and gay individuals was associated with decreased sexual prejudice and homonegativity among heterosexual individuals, and that even brief interactions fostered accepting attitudes toward lesbian and gay people.

It has been stated that the contact hypothesis is more complex than mere exposure and can be impacted by the type of contact, frequency, degree of closeness in the relationship, and the positivity/negativity of interactions^23^. Frias-Navarro et al.^24^ found that the relationship between the quantity of contact among the ingroup and the outgroup and the level of rejection of the outgroup is mediated by the quality of contact, specifically, their findings revealed the mediator role of satisfaction with contact, that is, a positive feeling and well-being when contact with same-sex parented families occurs. Following this trend, findings from a recent Australian study indicated that stronger bonds and closer relationships with sexual and gender minorities may be more like to significantly improve attitudes toward same-sex parenting^25^. We argue that the mechanism behind co-constructing positive and strong bonds with sexual and gender minorities is comfort with contact with LGB individuals. Comfort can be conceptualized as the positive affective response elicited within a relationship, providing ease that alleviates distress. Comfort functions to encourage contact between individuals and it garners positive attitudes toward the other person in a relationship. In Portugal, Costa et al.^26^ found that interpersonal contact with lesbian and gay individuals was linked to more favorable attitudes toward them as parents, and more specifically, that the association between interpersonal contact and attitudes toward same-sex parenting was mediated by comfort with lesbian and gay individuals, as the positive affect gained from contact evokes empathy toward them.

### Research on same-sex parenting in the majority world

Years of research in the Minority World^27^ have demonstrated that the individual differences associated with negative attitudes toward LGB people and same-sex parenting among cisgender heterosexual individuals overlap across studies and include being a man, in a relationship, having children, being older, religious, having a conservative political orientation and lacking a higher education degree^9,15,28^, as reviewed in the previous section. Even though this is a relatively recent and underexplored line of research in the Majority World^27^, which refers to countries widely spoken of as “developing” or “third world” nations of the “Global South”^29^, there is concrete evidence in Hispanic countries that follows the same direction. For example, Chilean university male students revealed higher levels of sexual prejudice toward same-sex parenting^30,31^ whereas younger, wealthier, and higher educated Colombian women were more likely to approve same-sex couple’s rights^32^. Ecuadorian men, heterosexual, those who practice their religion, those who attend more frequently to religious services, and those who identify as conservative showed higher levels of prejudice against lesbian and gay individuals as well as less support toward their rights, namely same-sex marriage and parenting^33^. Furthermore, in the same South American country, it was found that older participants, those who were married and those with children had less favorable attitudes toward same-sex parenting^34^. Costa and Salinas-Quiroz^14^ established that Mexican men, and religious participants showed higher levels of negative beliefs about same-sex parenting. Lastly, and at a larger scale—including 18 Latin American countries—it was discovered that people who embrace democracy and democratic values express greater support for same-sex marriage and that this decreased significantly when participants also take part in religious communities once a week or more frequently^35^. Nevertheless, more complex relationships between interpersonal contact and attitudes toward same-sex parenting, particularly the role of comfort with LGB people as mediator of the effects of interpersonal contact have not been investigated beyond the Minority World literature.

In the Majority World, Latin America is a collective term for the region where Romance Languages are predominantly spoken; it commonly refers to South America, Central America, Mexico, and the islands of the Caribbean^36^. As the present study focuses on Spanish-speaking countries in North, Central and South America, excluding the Caribbean, the term Continental American Hispanic Nations (CAHN) will be utilized henceforth. In CAHN, there are high levels of Catholic religiosity and its ties to conservatism^37^, familism, and gender-role traditionalism^14^—all of which are linked to negative attitudes toward same-sex parenting. In particular, the culture of *machismo*—in which men are expected to be hypermasculine, dominant, and aggressive—shapes the way gender roles and family values are delineated and instilled within CAHN societies^38^. Familism, or the prioritization of family above the individual, is characteristic of Hispanic culture and further contributes to the increased emphasis on traditional family dynamics^39^.

In the present study, we explored the effects of interpersonal contact and the mediator role of comfort with LGB people in explaining attitudes toward same-sex parenting beyond Anglo-Saxon and European nations. Mainly we aim to analyze the role of interpersonal contact and comfort in shaping attitudes toward same-sex parenting in the Majority World. CAHN are of particular interest given the dearth of research in this geographic area on attitudes toward same-sex parenting as well as the varying degrees of acceptance of and protections for same-sex parented families in each country. There is substantial evidence that legal progress and protection of LGB people’s rights is associated with greater accepting attitudes toward LGB people and same-sex marriage and parenting^40^. As shown in Table 1, the countries that constitute the Central region do not provide any legal recognition to LGB individuals and their families, except for Costa Rica that has recently approved both same-sex marriage and adoption by same-sex couples; the countries that compose the Amazonia region offer more legal protection to same-sex parented families as a whole and have started this process earlier than the Central region. The North region, composed only by Mexico, provides legal recognition to LGB individuals and same-sex parented families although the process has taken more than twelve years since the legislation must be approved independently by each state (32 in total). Lastly, the countries that compose the ConoSur region, particularly Argentina and Uruguay, have been leading legal progress in the CAHN geographic area.

**Table 1:** Same-sex marriage and parenting rights in some Continental American Hispanic Nations (CAHN) until February 2024.

Region	Country	Laws allowing same-sex marriage or civil union (year)	Adoption by same-sex parents (year)	Surrogacy for same-sex parents
North	Mexico	√ (2009–2022)	√ in 22/32 states	Only allowed when fertility issues affect the mother; forbidden for same-sex fathers
Central	Costa Rica	√ (2020)	√ (2020)	x
	El Salvador	x	x	x
	Guatemala	x	x	x
	Honduras	x	x	x
	Nicaragua	x	x	x
ConoSur	Argentina	√ (2010)	√ (2010)	Altruistic surrogacy is allowed; for-profit surrogacy is illegal
	Chile	√ (2022)	√ (2022)	√ (2022)
	Uruguay	√ (2013)	√ (2009)	Only allowed when (1) it is altruistic; (2) fertility issues affect the mother, thus forbidden for same-sex fathers
Amazonia	Bolivia	x—Civil unions allowed since 2020	x	Unregulated
	Colombia	√ (2016)	√ (2015)	Only allowed when (1) it is altruistic; (2) fertility issues affect the mother, thus forbidden for same-sex fathers
	Ecuador	√ (2019)	x	Unregulated
	Perú	x	x	Only allowed when fertility issues affect the mother; forbidden for same-sex fathers
	Venezuela	x	x	Altruistic surrogacy is allowed; for-profit surrogacy is illegal

However, with certain CAHN providing more legal protections for LGB people than others, it remains unclear whether these societal norms influence government policies and laws about same-sex parenting or vice versa^9,40^. Nevertheless, there is some evidence that changes in sexual prejudice arise from advancements in legal rights for sexual minorities^28^. The present study is the first to examine attitudes toward same-sex parenting among several CAHN, after previous research focused mostly on individual countries in the region such as Chile, Colombia, Ecuador or Mexico^14,30–34^.

### The present study

The objectives of the present study were threefold. The first objective was to explore the experiences of interpersonal contact and comfort with LGB people within four CAHN (North: Mexico; Central: Costa Rica, El Salvador, Guatemala, Honduras, and Nicaragua; ConoSur: Argentina, Chile, and Uruguay; and Amazonia: Bolivia, Colombia, Ecuador, Peru, and Venezuela). The second objective was to examine attitudes toward same-sex parenting across the four CAHN. The third objective was to examine the effects of sociodemographic variables and individual differences (age, gender, relationship status, parenthood status, level of education, religious beliefs, religiosity, and political leaning) and comfort with LGB people on attitudes toward same-sex parenting across the four CAHN.

## Methods

### Participants

The sample was composed by 1955 self-identified heterosexual cisgender participants from 14 CAHN; 782 from Mexico, 315 from Colombia, 252 from Argentina, 242 from Ecuador, 114 from Chile, 62 from Peru, 39 from El Salvador, 38 from Uruguay, 33 from Costa Rica, 19 from Guatemala, 18 from Nicaragua, 16 from Bolivia, 14 from Honduras, and 11 from Venezuela. Given the small number of cases from some of these countries and the unequal sample sizes, we decided to group the nations not only into their geopolitical regions but also taking into account levels of social acceptance of LGB people and their rights as well as legal frameworks that were put in place to recognize same-sex parenting (for more detailed information^40,41^). In the North region there is Mexico (*n* = 782; 40.0%), the Central region is composed by Costa Rica, El Salvador, Guatemala, Honduras, and Nicaragua (*n* = 123; 6.3%), and South America was divided into two regions; the south peninsula of ConoSur which included Argentina, Chile, and Uruguay (*n* = 404, 20.7%), and the Amazonia region which is comprised by Hispanic countries that contain the Amazon rainforest, i.e., Bolivia, Colombia, Ecuador, Peru, and Venezuela (*n* = 646, 33.0%). Within group differences (i.e., within each of the four regions) were investigated to safeguard the aggregation of countries and no significant nation-level differences on interpersonal contact experiences and attitudinal variables were found (comparison statistics are not included in the article but may be made available upon request).

Participants’ ages ranged from 18 to 87 (*M* = 35; *SD* = 12). Almost half of them were single and not in a committed relationship, and a slight majority reported not having children. The sample was highly educated, with about 80% reporting having a college degree, and the overwhelming majority was full-time employed, full-time student, or studying and working. Most participants identified as Hispanic, and a very small percentage of individuals (2%) reported being from an indigenous ethnical background. Almost half of the sample identified as Catholic, yet close to one fifth described themselves as being spiritual but not identifying with any organized religion, and under a third reported practicing their religion. Lastly, over 50% of participants proclaimed being on the left side of the political spectrum, and only 8% reported right-wing political leaning. Detailed sociodemographic data for the whole sample and separately for each region are presented in Table 2.

**Table 2:** Main sociodemographic data for the total sample and for each region separately.

	Total sample		Region							
			North		Central		ConoSur		Amazonia	
	*n* = 1955		*n* = 782		*n* = 123		*n* = 404		*n* = 646	
	Range	*M*(*SD*)	Range	*M*(*SD*)	Range	*M*(*SD*)	Range	*M*(*SD*)	Range	*M*(*SD*)
Age	18–87	35(12)	18–69	35(11)	18–63	32(11)	18–74	41(12)	18–87	31(12)
	*n*	%	*n*	%	*n*	%	*n*	%	*n*	%
Gender										
Man	546	28	331	42	32	26	32	8	151	23
Woman	1409	72	451	58	91	74	372	92	495	77
Relationship										
Yes	1025	52	439	56	54	44	291	72	241	38
Relationship status										
Single	930	48	343	44	69	56	113	28	405	63
Married	635	32	289	37	36	29	165	41	145	22
Civil partnership	231	12	95	12	10	8	78	19	48	7
Divorced	143	7	49	6	8	7	43	11	43	7
Widowed	16	1	6	1	0	0	5	1	5	1
Children										
Yes	929	48	386	49	46	37	299	74	198	31
Education										
College degree	1573	81	669	86	91	74	327	81	486	75
Occupation										
Student	343	17	76	9	38	31	18	5	211	33
Student & employed	271	14	101	13	16	13	41	10	113	17
Employed	778	40	343	44	35	28	215	53	185	29
Self-employed	370	19	190	24	23	19	70	17	87	13
Unemployed	168	9	68	9	10	8	43	11	47	7
Retired	25	1	4	1	1	1	17	4	3	1
Religion										
Catholic	928	47	429	55	51	41	116	29	332	51
Christian	161	8	34	4	22	18	30	7	75	12
Spiritual, not religious	430	22	162	21	27	22	116	29	125	19
Agnostic/Atheist	354	18	125	16	16	13	124	31	89	14
Other	82	5	32	4	7	6	18	4	24	4
Political leaning										
Extreme-left	228	12	99	13	11	9	61	15	57	9
Left	1021	52	482	62	52	42	194	48	293	45
Center-left	136	7	29	4	9	7	58	14	40	6
Center	93	5	28	4	9	7	20	5	36	6
Center-right	86	4	33	4	8	7	10	3	35	5
Right	46	2	9	1	6	5	0	0	31	5
Extreme-right	3	1	1	1	0	0	0	0	2	1

*Note* When percentages do not total 100 is due to missing values.

### Measures

Participants were asked to complete a series of sociodemographic questions, a questionnaire about their interpersonal contact/comfort experiences with LGB people, and the Attitudes toward Gay and Lesbian Parenting Scale.

#### Sociodemographic questionnaire

The sociodemographic questionnaire included sex assigned at birth (male/female/other), gender (men/women/other), sexual orientation (heterosexual/gay/lesbian/bisexual/other), being in relationship (yes/no), relationship status (categorical), having children (yes/no), level of education (categorical), current occupation (categorical), religious beliefs (categorical), religiosity (5-point Likert-type scale) and political leaning (7-point Likert-type scale).

#### Interpersonal contact and comfort with LGB people

Interpersonal contact experiences were measured using the interpersonal contact questions developed by Costa et al.^26^. Participants were asked “Do you have any?” (1) gay, lesbian and/or bisexual acquaintances, (2) friends, (3) family members, and (4) “Do you know any gay, lesbian and/or bisexual parented families?”, all measured on a “yes” or “no” format; and (5) “How comfortable do you feel with gay, lesbian and bisexual individuals?”, measured on a 5-point Likert-type scale ranging from 1 = "not comfortable at all" to 5 = "very comfortable", with higher scores reflecting greater comfort with LGB people.

#### Attitudes toward same-sex parenting

Attitudes toward Same-Sex Parenting were measured using the Attitudes toward Gay and Lesbian Parenting Scale^11^. This multidimensional scale consists of 11 items distributed into two factors: (1) Negative Perceptions of Gay and Lesbian Parenting, composed of six items (e.g., “Gay and lesbian parents do not care about the child’s best interest”; α = 0.84) and (2) Perception of Benefits of Gay and Lesbian Parenting, composed of five items (e.g., “The difficulties that gay and lesbian parents face prepare them to be good parents”; α = 0.70), with all items measured on a 5-point Likert-type scale (from 1 = "completely disagree" to 5 = "completely agree"). In the present study, negative attitudes toward Same-Sex Parenting was coded so that higher scores would reflect higher levels of negative attitudes whereas positive attitudes toward Same-Sex Parenting was coded so that higher scores would reflect higher levels of positive attitudes.

### Procedures

Data was collected between May and October 2019 through an online survey available from Qualtrics. The survey was administered in Spanish and was distributed by the research team, as well as advertised through their personal social networks (Facebook, Instagram, WhatsApp, and Twitter), and as college mailing lists, through non-probabilistic intentional and convenience sampling. To take part in the study, participants had to be at least 18 years old, be a Spanish speaker, and live in one CAHN. Before completing the survey, participants provided their consent, which was displayed on the first page. The informed consent stipulated the study’s objectives and conditions as well as information regarding possible risks and benefits. Participation was voluntary, anonymous, and individuals could withdraw from the study at any point. All research was performed in accordance with the ethical standards stated by 1964 Helsinki declaration and its later amendments or comparable ethical standards. The study protocol was reviewed and approved by the General Research Department from Universidad de Las Américas, Quito (Ecuador) and from the Ethics Committee, Universidad de Ibagué, Ibagué (Colombia).

## Results

### Interpersonal contact and comfort with LGB people

To evaluate interpersonal contact with LGB people, participants were asked if they had: (1) LGB acquaintances, (2) friends, (3) family members, and if they knew (4) a LGB parented family (Table 3). The overwhelming majority of participants in the whole sample reported having LGB acquaintances (98%) and friends (81%), although about half reported having an LGB family member and only about a third knowing an LGB-parented family. The mean score for comfort with LGB people was very high (4.35 in a 5-point scale). A standard multiple regression was developed to assess which interpersonal contact variables explained the levels of comfort with LGB people. The regression model was significant, *F*(4,1954) = 56.389, *p* < 0.001, R^2^_adj_ = 0.102, and only having an LGB family member was not significantly associated with levels of comfort (*p* = 0.262).

**Table 3:** Descriptive statistics and comparison statistics for interpersonal contact experiences and comfort with LGB People.

	Total sample		Region								Test results
			North		Central		ConoSur		Amazonia		
	*n* = *1955*		*n* = 782		*n* = 123		*n* = 404		*n* = 646		
	Yes	%	Yes	%	Yes	%	Yes	%	Yes	%	
LGB acquaintances	1920	98	772	99	122	99	400	99	626	97	χ^2^(3) = 9.567, *p* = 0.023
LGB friends	1583	81	658	84	89	72	322	80	514	80	χ^2^(3) = 12.279, *p* = 0.006
LGB family members	962	49	448	57	65	53	178	44	271	42	χ^2^(3) = 38.982, *p* < 0.001
LGB parented families	664	34	254	33	36	29	193	48	181	28	χ^2^(3) = 46.820, *p* < 0.001
	*M*	*SD*	*M*	*SD*	*M*	*SD*	*M*	*SD*	*M*	*SD*	*F*_w_(3,506.347) = 25.429, *p* < 0.001
Comfort with LGB people	4.35	0.97	4.36	0.94	4.32	1.00	4.64	0.75	4.15	1.07	

To compare the prevalence of interpersonal contact with LGB people comparatively across the four CAHN regions, Chi-square tests were conducted. Group differences were found for all four interpersonal contact variables (Table 3). Individuals from the Amazonia region were less likely to have LGB acquaintances whereas participants from the Central region were less likely to have LGB friends than people from the other regions. Participants from the North region were more likely to report having a LGB family member. Lastly and noteworthy, participants from the ConoSur region were more likely to know LGB parented families, followed by participants from the North region.

Given the violation to the homogeneity of variances, one-way ANOVAs with Welsh correction were conducted. Further, Games-Howell post-hoc tests were conducted for pairwise comparisons which are recommended when equal variances cannot be assumed. Regarding the level of comfort with LGB people, significant group differences were found following a one-way ANOVA with Welsh correction. Games-Howell post-hoc tests showed that participants from the ConoSur region reported the highest level of comfort with LGB people compared to the other regions (*p*’s < 0.01), followed by participants from the North region when compared to the Amazonia region (*p* = 0.001), but not significantly different from the Central region (*p* = 0.969). Participants from the Amazonia and Central regions did not significantly differ in their reported comfort with LGB people (*p* = 0.366).

Four standard multiple regressions were conducted to assess the relative importance of interpersonal contact experiences on comfort with LGB people, separately for each region (Table 4). For the North region, the regression model was significant, *F*(4,781) = 15.545, *p* < 0.001, *R*^2^_adj_ = 0.069, and a significant portion of the variance of comfort with LGB people was explained by having LGB acquaintances and friends, but not by knowing a LGB parented family nor by having a LGB family member. For the Central region, the regression model was significant, *F*(4,122) = 2.612, *p* = 0.039, *R*^2^_adj_ = 0.050, and a significant portion of the variance of comfort with LGB people was explained only by having LGB friends. For the ConoSur region, the regression model was significant, *F*(4,403) = 3.580, *p* = 0.007, R^2^_adj_ = 0.025, and a significant portion of the variance of comfort with LGB people was explained only by having LGB friends. Lastly, for the Amazonia region, the regression model was significant, *F*(4,645) = 36.826, *p* < 0.001, *R*^2^_adj_ = 0.182, and a significant portion of the variance of comfort with LGB people was explained only by having LGB acquaintances and friends. Taken together, these results showed that having LGB friends was the only significant interpersonal contact variable across the four regions.

**Table 4:** Regression analyses for the effects of interpersonal contact on comfort with LGB people.

	Total sample				Region															
					North				Central				ConoSur				Amazonia			
	*n* = *1955*				*n* = 782				*n* = 123				*n* = 404				*n* = 646			
	B	β	*t*	*p*	B	β	*t*	*p*	B	β	*T*	*p*	B	β	*t*	*p*	B	β	*t*	*p*
LGB acquaintances	0.859	0.117	5.306	< 0.001	0.640	0.076	2.130	0.034	− 0.194	− 0.017	− 0.195	0.846	0.202	0.027	0.533	0.594	0.907	0.147	3.948	< 0.001
LGB friends	0.588	0.238	10.557	< 0.001	0.565	0.219	6.031	< 0.001	0.497	0.223	2.424	0.017	0.246	0.131	2.589	0.010	0.896	0.338	8.845	< 0.001
LGB family members	0.048	0.025	1.122	0.262	0.020	0.010	0.293	0.770	0.276	0.138	1.508	0.134	− 0.075	− 0.049	− 1.002	0.317	0.105	0.048	1.291	0.193
LGB parented families	0.179	0.087	3.995	< 0.001	0.136	0.068	1.923	0.055	0.012	0.005	0.060	0.952	0.143	0.095	1.890	0.059	0.135	0.057	1.540	0.122

### Attitudes toward same-sex parenting

The mean scores on negative attitudes toward same-sex parenting were below the scale’s midpoint whereas the mean scores on positive attitudes toward same-sex parenting were above the scale’s midpoint, suggesting overall positive attitudes toward same-sex parenting. The correlation between the two variables was moderate and significant (*r* =  − 0.464, *p* < 0.001). To compare the four CAHN regions on negative attitudes toward same-sex parenting and positive attitudes toward same-sex parenting two one-way ANOVAs were conducted. No severe violations to the normality assumption were found, although group variances were found not to be homogeneous (Table 5).

**Table 5:** Descriptive statistics for negative attitudes toward Same-Sex Parenting (SSP) and positive attitudes toward Same-Sex Parenting (SSP).

	Total sample		Region								Test results
			North		Central		ConoSur		Amazonia		
	*n* = *1955*		*n* = 782		*n* = 123		*n* = 404		*n* = 646		
	*M*	*SD*	*M*	*SD*	*M*	*SD*	*M*	*SD*	*M*	*SD*	
Negative attitudes SSP	2.16	0.74	1.55	0.63	1.79	0.89	1.36	0.53	1.81	0.86	*F*_W_(3,498.922) = 78.384, *p* < 0.001
Positive attitudes SSP	3.60	0.60	2.15	0.67	2.38	0.82	1.78	0.53	2.38	0.83	*F*_W_(3,495.869) = 4.828, *p* = 0.003

Significant group differences were found regarding negative attitudes toward same-sex parenting, *F*_W_(3,498.922) = 78.384, *p* < 0.001, η_p_^2^ = 0.088. Pairwise comparisons revealed significant differences between all regions (all *p*’s < 0.05) except for Central versus Amazonia regions (*p* = 1.00). The ConoSur region displayed the lowest negative attitudes toward same-sex parenting, followed by the North region, and with similar negative attitudes in the Central and Amazonia regions. Regarding the positive attitudes toward same-sex parenting, significant group differences were also found, *F*_W_(3,495.869) = 4.828, *p* = 0.003, η_p_^2^ = 0.008. Pairwise comparisons revealed significant differences between most groups (all *p*’s < 0.05) except for Central versus ConoSur regions (*p* = 0.430), and between Amazonia versus North and Amazonia versus ConoSur regions (*p* = 149; *p* = 0.969; respectively).

### Comfort with LGB people and attitudes toward same-sex parenting

Two hierarchical multiple regression analyses were conducted to examine if participants’ sociodemographic characteristics and comfort with LGB people would explain the levels of negative attitudes toward same-sex parenting and the positive attitudes toward same-sex parenting (Table 6). Sociodemographic variables were introduced in the first step and comfort with LGB people was introduced in the second step. In the first regression model, the first step was significant, *F*(8,1596) = 52.285, *p* < 0.001, R^2^_adj_ = 0.204, and gender, level of education, being religious, religiosity, and political leaning were significantly associated with negative attitudes toward same-sex parenting; Age, being in a relationship, and having children were not. The second step was also significant, *F*(9,1596) = 95.289, *p* < 0.001, R^2^_adj_ = 0.347, and added a significant proportion of explained variance, *F*_change_(1,1587) = 347.936, *p*_change_ < 0.001. Comfort with LGB people was significantly associated with negative attitudes toward same-sex parenting after controlling for the effects of sociodemographic characteristics (Table 6).

**Table 6:** Regression analyses for the effects of sociodemographic characteristics and comfort with LGB people on negative attitudes toward Same-Sex Parenting (SSP) and positive attitudes toward Same-Sex Parenting (SSP).

	Total sample							
	Negative attitudes SSP				Positive attitudes SSP			
	B	β	*t*	*p*	B	β	*t*	*p*
Step 1								
Age	0.000	0.005	0.186	0.853	− 0.002	− 0.044	− 1.456	0.146
Gender	0.270	0.158	6.857	< 0.001	− 0.212	− 0.154	− 6.250	< 0.001
Relationship	0.027	0.018	0.588	0.557	− 0.077	− 0.064	− 1.938	0.053
Children	0.040	0.027	0.848	0.397	− 0.060	− 0.051	− 1.485	0.138
Education	− 0.202	− 0.097	− 4.254	< 0.001	− 0.019	− 0.011	− 0.466	0.641
Religion	− 0.317	− 0.168	− 7.130	< 0.001	0.065	0.043	1.708	0.088
Religiosity	0.145	0.314	13.340	< 0.001	− 0.074	− 0.199	− 7.905	< 0.001
Political leaning	0.051	0.080	3.420	< 0.001	− 0.020	− 0.039	− 1.578	0.115
Step 2								
Comfort with LGB people	− 0.313	− 0.406	− 18.653	< 0.001	0.153	0.248	9.912	< 0.001

In the second regression model, the first step was significant, *F*(8,1596) = 19.553, *p* < 0.001, R^2^_adj_ = 0.085, and gender and religiosity were significantly associated with positive attitudes toward same-sex parenting, but being in a relationship and an being religious were marginally significant; age, level of education, political leaning and having children were not. The second step was also significant, *F*(9,1596) = 29.361, *p* < 0.001, R^2^_adj_ = 0.138, and added a significant proportion of explained variance, *F*_change_(1,1587) = 347.936, *p*_change_ < 0.001. Comfort with LGB people was significantly associated with positive attitudes toward same-sex parenting after controlling for the effects of sociodemographic characteristics.

Lastly, two hierarchical multiple regression analyses were conducted for each region to examine if participants’ sociodemographic characteristics and comfort with LGB people would explain the levels of negative attitudes toward same-sex parenting and positive attitudes toward same-sex parenting (Table 7). Regarding negative attitudes toward same-sex parenting, some differences were found across the four CAHN regions, although a relevant trend was found insofar as religiosity was a significant predictor of negative attitudes for all regions, with a very high beta level for the Central region. Gender was only significant for the North and Amazonia regions and political leaning only for the North region. Of note, being in a relationship and having children was not significant for any of the regions. Lastly, comfort with LGB people was significantly explained the levels of negative attitudes in all four regions after controlling for the effects of the sociodemographic variables. Regarding positive attitudes toward same-sex parenting, the findings varied greatly across regions, with religiosity being negatively associated with positive attitudes toward same-sex parenting for the North, Central, and Amazonia regions but not for the ConoSur region. Gender was also a significant predictor for the North and Amazonia regions and being in a relationship was also significant for the North region and marginally significant for the ConoSur region. After controlling for the effects of the sociodemographic variables, comfort with LGB people was only significant for the North and Amazonia regions.

**Table 7:** Regression analyses for the effects of sociodemographic characteristics and comfort with LGB people on negative attitudes toward Same-Sex Parenting (SSP) and positive attitudes toward SP, separately for CAHN.

	CAHN															
	North				Central				ConoSur				Amazonia			
	*n* = 782				*n* = 123				*n* = 404				*n* = 646			
	B	β	*t*	*p*	B	β	*t*	*p*	B	β	*t*	*p*	B	β	*t*	*p*
Negative attitudes SSP																
Step 1																
Age	0.005	0.091	2.116	0.035	− 0.002	− 0.019	− 0.168	0.867	− 0.001	− 0.015	− 0.253	0.801	0.002	0.024	0.420	0.675
Gender	0.221	0.158	3.992	< 0.001	0.091	0.047	0.505	0.615	0.101	0.055	1.009	0.314	0.258	0.130	3.138	0.002
Relationship	0.022	0.016	0.347	0.729	− 0.066	− 0.038	− 0.312	0.756	− 0.052	− 0.048	− 0.774	0.440	0.131	0.077	1.246	0.213
Children	0.089	0.066	1.365	0.173	0.203	0.114	0.972	0.334	0.083	0.074	1.141	0.255	0.101	0.057	0.916	0.360
Education	− 0.155	− 0.075	− 2.069	0.039	− 0.214	− 0.095	− 0.994	0.323	− 0.177	− 0.135	− 2.497	0.013	− 0.308	− 0.143	− 3.406	< 0.001
Religion	− 0.358	− 0.197	− 5.298	< 0.001	− 0.119	− 0.046	− 0.480	0.633	− 0.069	− 0.068	− 1.194	0.233	− 0.362	− 0.151	− 3.485	< 0.001
Religiosity	0.086	0.192	5.219	< 0.001	0.224	0.492	5.118	< 0.001	0.069	0.162	2.915	0.004	0.151	0.331	7.776	< 0.001
Political leaning	0.094	0.141	3.565	< 0.001	0.048	0.077	0.832	0.408	0.037	0.069	1.246	0.214	0.006	0.010	0.233	0.816
Step 2																
Comfort with LGB people	− 0.250	− 0.344	− 9.813	< 0.001	− 0.173	− 0.208	− 2.090	0.040	− 0.150	− 0.234	− 4.386	< 0.001	− 0.410	− 0.528	− 13.912	< 0.001
Positive attitudes SSP																
Step 1																
Age	0.000	0.005	0.112	0.911	− 0.005	− 0.072	− 0.608	0.544	− 0.004	− 0.099	− 1.619	0.106	− 0.002	− 0.034	− 0.581	0.561
Gender	− 0.174	− 0.143	− 3.386	< 0.001	− 0.175	− 0.106	− 1.088	0.280	− 0.033	− 0.018	− 0.316	0.752	− 0.332	− 0.218	− 5.116	< 0.001
Relationship	− 0.136	− 0.116	− 2.307	0.021	− 0.260	− 0.174	− 1.381	0.171	0.121	0.108	1.697	0.091	− 0.105	− 0.080	− 1.268	0.206
Children	− 0.039	− 0.033	− 0.647	0.518	0.009	0.006	0.048	0.962	− 0.087	− 0.076	− 1.139	0.255	− 0.088	− 0.065	− 1.013	0.312
Education	− 0.079	− 0.044	− 1.136	0.256	0.048	0.025	0.251	0.802	− 0.055	− 0.041	− 0.737	0.462	0.079	0.048	1.113	0.266
Religion	0.125	0.080	2.003	0.046	0.043	0.019	0.196	0.845	− 0.039	− 0.037	− 0.645	0.519	0.097	0.053	1.185	0.236
Religiosity	− 0.045	− 0.117	− 2.965	0.003	− 0.097	− 0.248	− 2.488	0.015	− 0.019	− 0.043	− 0.748	0.455	− 0.105	− 0.300	− 6.857	< 0.001
Political leaning	− 0.017	− 0.029	− 0.694	0.488	− 0.167	− 0.312	− 3.249	0.002	− 0.003	− 0.006	− 0.110	0.912	0.002	0.005	0.118	0.906
Step 2																
Comfort with LGB people	0.167	0.265	6.819	< 0.001	0.079	0.112	1.064	0.291	0.013	0.019	0.340	x	0.195	0.327	7.464	< 0.001

Taken together, these findings suggest that comfort with LGB people was important in explaining negative attitudes toward same-sex parenting across all four CAHN regions, over and beyond other sociodemographic variables such as gender or religiosity. Yet, comfort with LGB people was only important for explaining the positive attitudes toward same-sex parenting in the North and Amazonia regions.

## Discussion

The present study explored the effects of interpersonal contact and the mediator role of comfort with LGB people in explaining attitudes toward same-sex parenting in 1955 heterosexual cisgender participants from 14 Continental American Hispanic Nations (CAHN; i.e., North, Central, Amazonia and Cono Sur). Firstly, the experiences of interpersonal contact and comfort with LGB people within four CAHN were explored; secondly, the attitudes toward same-sex parenting across the fours CAHN were examined; and lastly, the effects of sociodemographic variables and individual differences and comfort with LGB people on attitudes toward same-sex parenting across the four CAHN were explored.

The results uncovered variability in the levels of contact with LGB people as well as attitudes toward same-sex parenting. Notably, knowing families headed by LGB parents and having a LGB family member were most commonly found among individuals from the ConoSur and North regions. Correspondingly, participants from these regions displayed the lowest levels of negative attitudes toward same-sex parenting. Participants from the Amazonia and Central regions displayed the lowest levels of contact with LGB people and the highest levels of negative attitudes toward same-sex parenting. As such, a relationship among contact with sexual minoritized individuals and attitudes toward same-sex parenting exists insofar as higher levels of contact and comfort with LGB persons were associated with lower levels of negative attitudes toward same-sex parenting, and in some regions, with higher levels of positive attitudes toward same-sex parenting.

International evidence, particularly that from the Minority World (e.g., Europe, US), supports this relationship between contact and attitudes and its extrapolation across cultures and contexts^9,22,24,26^. Nevertheless, while comfort level varied by region in a pattern similar to contact level, our results demonstrated the role of comfort explains a relevant level of the variance of attitudes toward same-sex parenting, transcending regional variation and extending across CAHN. Regarding the relationship between interpersonal contact and attitudes toward same-sex parenting, the variations between the four different CAHN regions were expected given the vast differences in legal protections across this geographic area. As shown in Table 1, the ConoSur includes the first two countries—Argentina and Uruguay—to legalize both same-sex marriage and adoption by LGB individuals. Consequently, the number and social visibility of LGB parented families have risen in these countries for around a decade prior to this study. Further, in the ConoSur we found a noteworthy pattern of findings regarding attitudes toward same-sex parenting; whilst the levels of negative attitudes toward same-sex parenting was found to be the lowest in this region when compared to the other CAHN, the levels of perception of benefits regarding same-sex parenting were also found to be lowest. This pattern of findings seems to suggest that more liberal attitudes in the ConoSur region, particularly in Argentina and Uruguay, may come from a normalization process of same-sex rights, namely, marriage and parenting. Despite legal progress not being the only indicator of social attitudes, it is an important one, and given the time elapsed since same-sex marriage and parenting were first legalized, there seems to be an integration of acceptance of LGB rights in society, what we call *embraced equality.* While negative attitudes toward same-sex parenting are low so are the perception of specific benefits associated with same-sex parenting comparing to different-sex parenting.

However, also within the ConoSur there is Chile, in which same-sex marriage and same-sex parenting had not yet been legalized at the time this study was conducted. As a result, we cannot conclude that the high levels of contact with LGB families in participants from the ConoSur is solely consequent to the region’s most protective legal advancements and more accepting attitudes toward LGB people and their families. One potential factor contributing to this result is the sociodemographic characteristics of our sample: the vast majority of participants were highly educated and did not endorse right-wing conservative political orientation. Consequently, the sample may not accurately reflect the demographics within CAHN, possibly contributing to a higher prevalence of contact with LGB parented families in the ConoSur. However, while there may be subtle differences between the three countries that compose the ConoSur region, we found no significant differences between the samples from Argentina, Chile, and Uruguay.

Within the North region, Mexico’s legal protections are regulated at the state level, giving rise to uneven degrees of protections for, contact with, and attitudes toward LGB people and same-sex parenting across the country. Mexico City in particular is arguably one of the most progressive megalopolis within CAHN, approving sweeping protections of LGB individuals in 2009^42^. However, until 2023 several states (i.e., Durango, Guerrero, Jalisco, Estado de México, Tabasco, Tamaulipas, Veracruz, and Yucatán) had deficiencies of any legal protections for sexual and gender minoritized individuals, potentially attributed to the tight grip Catholicism holds on the culture of these states that stigmatizes LGB people with its heterosexist values^14^ and compulsory heterosexuality^7^. Nevertheless, the results demonstrated high levels of contact with and accepting attitudes toward LGB individuals and same-sex parenting, which can be at least partially attributed to decades of queer activism nationwide.

Central and Amazonia regions are the least protective of LGB rights within CAHN, so the lowest levels of contact and higher negative attitudes were unsurprisingly found within these regions. Although both contain one or more countries that afford same-sex couples some legal rights, most countries in the Central and Amazonia regions fail to adequately validate and support LGB individuals through legal protections, corresponding to high levels of negative attitudes toward them and same-sex parenting. As a result, LGB people in some of these countries might compartmentalize and hide parts of themselves or conceal their sexual orientation to avoid the consequences of stigma and discrimination, since expressing their identity and/or orientation might carry individual and interpersonal costs that LGB individuals may believe are better to avoid. However, it is important to note that despite commonalities between the countries that compose these two regions in respect to legal protection of LGB rights, the countries are evolving at different paces and the pedagogical role of the law in promoting social change takes time. Zooming in on the specific findings regarding these two regions, we found hardly any differences between them on comfort with LGB people and attitudes toward same-sex parenting. However, these findings should be read with caution: The Central region was composed by only 123 invididuals in this study, with very few participants in each country. In addition, the large sample size from Amazonia mostly comprises participants from Colombia (*n* = 315) and Ecuador (*n* = 114), arguably, the most progressive in the region regarding LGB rights, but where the legal achievements are very recent.

Regarding the role of comfort in shaping attitudes, our results corroborate and expand previous European studies^24,26^ across all four CAHN regions: comfort was vital in decreasing negative attitudes toward same-sex parenting but not necessarily in supporting the positive attitudes, i.e., the perception of benefits associated with same-sex parenting. These findings are remarkable in that they transcend the aforementioned pattern in contact and attitudes—regardless of differences in the legal and social climate for LGB people and parents, comfort is crucial in yielding wider attitudinal change among CAHN. Because the impact of comfort on reducing negative attitudes toward same-sex parenting was significant across CAHN, interventions that increase comfort can be effective throughout the regions.

The pattern of levels of comfort with LGB people similarly resembled those of both contact and attitudes as previously mentioned, with participants from the ConoSur and North regions reporting the most elevated comfort levels. However, the magnitude of the impact of comfort in reducing negative attitudes differed among CAHN. Within the Central and Amazonia regions, increased comfort was associated with more strongly reduced negative attitudes, whereas the importance of comfort was weaker for negative attitude reduction in the North and ConoSur. These findings suggest that comfort with LGB people has a particularly powerful influence on reducing negative attitudes toward same-sex parenting in areas with less legal and cultural acceptance of LGB individuals. For example, the Central and Amazonia regions predominantly consist of countries in which sexual and gender minoritized individuals are not protected or validated, so increasing comfort with LGB people may foster more accepting attitudes toward them at a regional scale. It is likely that, in these regions, relationships with LGB individuals will not form easily, given the polarized political and attitudinal climate.

Interventions are needed to foster positive connections with LGB people. For example, the school system is an impactful locus of change, in which families can come together and create relationships with same-sex parents and LGB individuals, ultimately reducing sexual prejudice. However, interventions can and should be conceptualized beyond schools as well to incorporate wider, mutually affirming engagement between LGB and heterosexual individuals. Given the sweeping positive impact of comfort demonstrated in each region, such interventions can be implemented across CAHN to encourage broader societal acceptance of LGB people and same-sex parenting. There is hope, since a recent study found that even a brief educational intervention that presents data about same-sex parenting—one page of written text containing evidence-based information—can have a positive impact on negative attitudes toward same-sex parented families^43^.

The findings suggest that Costa et al.’s^26^ model can be applied in the Majority World to encompass a wide variety of CAHN. The role of comfort in shaping negative attitudes supersedes cultural differences—with the familistic, *machista* CAHN contrasting the more individualistic USA^44^. Because the model is not limited to Europe and the USA, our study confirms that comfort should be further investigated around the world, providing a potentially global framework to assess comfort and sexual attitudes. The present study also puts forward the scope of comfort’s political implications: with vastly different sociocultural climates within CAHN, as well as the rise of populism and Christian extremist groups, comfort can serve as a common foundation to collectively improve attitudes.

Germane to study limitations, the sample overall may not have accurately reflected the CAHN population. In this study, only 2% of the sample reported being from an indigenous ethnical background. The low representation of indigenous people impacts the generalizability of the findings and may not adequality capture the variations that could exist within individuals from these communities. The limited participation of indigenous individuals also hints at potential barriers that warrant exploration in future studies. Specifically, considerations for cultural and linguistic sensitivity should be prioritized, perhaps by adapting questionnaires to languages such as Quechua, Nahuatl, Aymara, or Mayan, to name a few. Moreover, the study signals the importance of revisiting recruitment strategies that ensure participation of individuals from various ethnic backgrounds (e.g., respondent-driven sampling), who are not easily engaged through online surveys.

In addition, most participants were highly educated—a sociodemographic factor associated with more positive attitudes toward LGB people and same-sex parenting. With higher levels of education, respondents likely possessed greater knowledge about or sensitivity toward the topics of stereotypes and prejudice. Furthermore, because the percentage of Catholic participants was below 50%, our sample does not represent CAHN religious environment: according to Pew Research Center^45^, nearly 70% of Latin Americans practice Catholicism. As a result, future studies should employ representative sampling to encapsulate the state of attitudes toward LGB people and same-sex parenting. Another important limitation regards a greater representation of women in the sample, who are traditionally more accepting of gender and sexual minoritized individuals and their rights. Further, the sample in this study was heterogenous but unequal for each of the nations included. These limitations are consequent to the sampling procedures employed and possible self-selection biases, and common among online surveys. To the extent that these possible biases have influence the results is unknown, but we can assume that the overrepresentation of women may have enhanced a more positive stance regarding same-sex parenting. Lastly, further research should incorporate more comprehensive assessments of comfort beyond a single item. We recommend a mixed methods approach to understand how comfort is manifested among individuals.

## Conclusions

Our results beg the question: do advancements in legal LGB protections shape attitudes toward same-sex parenting, or vice versa? Based on the breakdown of the four regions, the answer is inconclusive given the legal variety within CAHN. All four regions consist of countries that have limited or no legal protections. For instance, until November 2021, Chile had no legal protections but is located in a region found to have high contact with LGB people and low levels of negative attitudes toward same-sex parenting; in countries like Colombia, the opposite was detected. These examples demonstrate a deviation from the pattern found by Bettinsoli et al.^28^, who concluded that attitudes toward LGB people and their rights were influenced by the country’s legal landscape. In sum, although region-based trends of contact with and attitudes toward LGB people and same-sex parenting are evident, we must take into consideration the variety within the CAHN geographic area; even though the four regions are territorially divided, it is integral that we account for the uneven state of protections that do not abide by location.

Policies are not enough to instill widespread change: we must encourage, facilitate, and supervise the formation of relationships with LGB individuals. Knowledge in the field of attitudes toward LGB people and same-sex parenting is primarily rooted in world trends and politics, but Allport’s (1954) seminal work on contact and Costa et al.’s^26^ foundational model on comfort ground the field in human interactions. The present study is the first to conduct research on comfort with LGB people and attitudes toward same-sex parenting across a Majority World region with great political and social diversity, demonstrating the significant association between increasing comfort and more accepting attitudes toward same-sex parenting across CAHN. Although we cannot conclude whether laws shape attitudes or vice versa^40^, it is clear that accepting attitudes can be shaped through fostering relationships. The amelioration of comfort with LGB people and same-sex parenting can predict societal improvements in attitudes, justifying the need for interpersonal interventions.

## Data Availability

The datasets generated during and/or analyzed during the current study are available from the corresponding author on a reasonable request.
